# Effects of smoking behavior on lung metastasis in the All of Us Research Program

**DOI:** 10.1038/s41598-025-89209-4

**Published:** 2025-04-01

**Authors:** Vikram R. Shaw, Jinyoung Byun, Younghun Han, Christopher I. Amos

**Affiliations:** 1 Institute for Clinical and Translational Research, Baylor College of Medicine, Houston, TX USA; 2 Section of Epidemiology and Population Sciences, Department of Medicine, Baylor College of Medicine, Houston, TX USA; 3 University of New Mexico Comprehensive Cancer Center, Albuquerque, NM 87131 USA

**Keywords:** Cancer epidemiology, Cancer prevention

## Abstract

Smoking may be associated with an increased risk of lung metastasis in cancers of non-lung origin. We leverage survey and electronic health record data from the diverse All of Us Research Program (AoURP) database to investigate whether smoking and smoking-related behaviors increase the risk of lung metastasis in non-lung primary cancers. The results suggest that cigarette use, measured by four continuous variables, does not increase the risk of lung metastasis in seven common cancer types but demonstrates a small significant effect in a cohort including all types of cancer in the database in both univariable and multivariable analyses. An increased odds ratio of electronic smoke use in patients with lung metastasis was seen in multivariable analyses of the all cancer (OR = 1.29, 95% CI = 1.04–1.59, *P* = 0.02) and liver cancer (OR = 1.57, 95% CI = 1.06–2.28, *P* = 0.02) groups. After adjusting for estimated cigarette pack years in the multivariable model, the result remained significant for liver cancer (OR = 1.60, 95% CI = 1.02–2.47, *P* = 0.04) but not the all cancer cohort. These results warrant further inquiry and suggest that smoking and e-cigarettes may be associated with lung metastasis risk in patients with non-lung tumors.

**Supplementary Information:**

The online version contains supplementary material available at 10.1038/s41598-025-89209-4.

## Introduction

Cancer is the second leading cause of death in the U.S., responsible for 600,000 deaths in 2020^1^. One of the primary drivers of cancer mortality is metastasis, which can lead to the disruption in the function of organs and organ systems. The metastasis of cancers of non-lung origin to the lung has been well described, and it is thought that organ-specific metastasis relies on a pre-metastatic niche (PMN) in the target secondary organ^2^. The formation of a lung PMN is a complex process that results from immune cells in the surrounding microenvironment and crosstalk between the primary tumor and metastatic cells, among other involved components^2^.

Studies have demonstrated that smoking may be associated with an increased risk of lung metastasis in cancers of non-lung origin. For example, in breast cancer, evidence suggests that a nicotine-promoted proinflammatory microenvironment may play an important role in metastasis^3–5^. Specifically, chronic nicotine exposure recruits pro-tumor N2-neutrophils, which induce the mesenchymal-epithelial transition and facilitate tumor colonization and metastasis^5^. In pancreatic cancer, smoking has been shown to promote metastasis into the liver and lung^6^. In colorectal cancer, multivariate analysis demonstrates that being a current smoker was an independent risk factor for pulmonary metastases^7^. In addition to the role of cigarette smoking, e-cigarettes, which have dramatically increased in popularity in recent years^8^, have been shown to promote breast carcinoma progression and lung metastasis^9^. While the effect of e-cigarettes on lung metastasis is understudied, another paper suggests that non-nicotine e-cigarettes may promote liver fibrosis, suggesting that e-cigarettes may alter underlying tissue integrity^10,11^. These results suggest that a large-scale characterization of smoking-related behavior on lung metastasis across common cancer types may provide insights that help guide future prospective studies and experimental inquiry that may ultimately influence clinical screening and treatment decisions. We hypothesize that smoking and smoking-related behaviors may play a role in the establishment of the lung PMN and metastasis of tumors of non-lung origin to the lung. In the present study, we utilize data from the National Institutes of Health’s All of Us Research Program (AoURP) and perform univariable and multivariable analyses to characterize the association between smoking and smoking-related behaviors and lung metastasis.

## Methods

The AoURP is a prospective cohort study with the objective of recruiting > 1 million US individuals to provide a comprehensive database for researchers interested in questions related to participant lifestyle, access to care, environment, and genomics^12^. Data in the AoURP is collected through self-reported surveys, physical wearables, and electronic health records (EHR). Using the cohort builder function within the *All of Us* workbench, we created each case-specific cohort for patients with breast, liver, colorectal, ovarian, pancreatic, prostate, and renal cancer, respectively, in addition to a case cohort of patients with all cancer types in the database and lung metastasis. The all cancer group includes the seven previously mentioned common cancer types, in addition to all other listed cancers in the database, except primary lung cancer. All cancer-related diagnoses for the case cohorts were extracted from EHR data, while analysis of smoking-related behaviors was obtained from survey data that were collected at the time of study enrollment.

We analyzed the relationship between various smoking-related variables and lung metastasis in breast, liver, colorectal, ovarian, pancreatic, prostate, and renal cancer using data from participants with these and other cancer types from the AoURP. We first investigated quantitative survey parameters, including the number of years smoked, the current number of cigarettes smoked per day, the average number of cigarettes smoked per day, and the estimated cigarette pack years across the seven cancer types, followed by an analysis of binary survey outcomes related to cigarette and smoking-related behaviors. We also performed our analyses in the all cancer cohort, which included all other listed cancers in the database, except primary lung cancer in order to control for overestimation of risk that might be associated with lung cancer recurrences after primary lung cancer. Significance was established for all analyses using logistic regression, with *P* < 0.05 considered significant. Multivariable logistic regression models were adjusted for age, sex, income, and insurance status (sex not included for ovarian and prostate cancer analyses). Participants who listed “Not male, not female, prefer not to answer, or skipped” or “No matching concept” for sex at birth and “Don’t Know/Prefer Not To Answer/Skip” for insurance or income were excluded from the multivariable analysis. The estimated cigarette pack years were calculated by multiplying a number of years smoking in the past by the average number of cigarettes smoked per day divided by twenty.

Informed participant consent was obtained by the AoURP, with all participants consenting to participate, viewing information about the AoURP goals, and what participation entails, and full details on the consent process are available through the AoURP at this link: https://allofus.nih.gov/about/protocol/all-us-consent-process. Analytic methods were carried out in accordance with relevant guidelines and regulations, in addition to adhering to AoURP policies. No experimental protocols were included in this study and additional ethical approval was not required as the data utilized in this study was de-identified.

## Results

The patient demographic data and EHR Systemized Nomenclature of Medicine (SNOMED) codes are shown in Table 1. The highest rate of lung metastasis was observed in liver cancer (21.9%) followed by pancreatic (9.7%) and renal (9.7%) cancers. The lowest rate of lung metastasis was observed in prostate cancer (1.9%), followed by breast cancer (2.8%). Univariable and multivariable analyses of the number of years smoked, the current number of cigarettes smoked per day, the average number of cigarettes per day, and the estimated cigarette pack years demonstrated no statistically significant differences between patients with and without lung cancer metastasis in the seven studied cancer types, except for a significant weakly negative effect (*P* = 0.01) of number of years smoked on liver cancer metastasis to the lung in the univariable analysis (Table 2). In the all cancer case group, however, a significant weakly positive effect was observed for all four variables.

**Table 1:** Demographic information for participants included in the study.

	Breast (*n* = 7,210)	Colorectal (*n* = 2,239)	Liver (*n* = 1,567)	Ovarian (*n* = 939)	Pancreatic (*n* = 662)	Prostate (*n* = 4,702)	Renal (*n* = 1,416)	All cancer** (*n* = 37,118)
Sex (female)	7,106 (98.6%)	1,203 (53.7%)	825 (52.6%)	932 (99.3%)	350 (52.9%)	50 (1.1%)	587 (41.5%)	21,312 (57.4%)
Race								
*White*	5,278 (73.2%)	1,582 (70.7%)	1,036 (66.1%)	672 (71.6%)	470 (71.0%)	3,572 (76.0%)	982 (69.4%)	27,629 (74.4%)
*Black or African * *American*	848 (11.8%)	297 (13.3%)	251 (16.0%)	101 (10.8%)	89 (13.4%)	625 (13.3%)	222 (15.7%)	4,393 (11.8%)
*Asian*	175 (2.4%)	43 (1.9%)	29 (1.9%)	23 (2.4%)	< 20*	44 (0.9%)	< 20*	593 (1.6%)
* Other*	909 (12.6%)	317 (14.2%)	251 (16.0%)	143 (15.2%)	> 20*	461 (9.8%)	> 20*	4,503 (12.1%)
Ethnicity (Hispanic or Latino)	692 (9.6%)	246 (11.0%)	193 (12.3%)	109 (11.6%)	67 (10.1%)	265 (5.6%)	171 (12.1%)	3,412 (9.2%)
Age (median, IQR)	70 (60–77)	71 (63–78)	69 (61–75)	67 (57–74)	72 (63–78)	76 (71–81)	71 (62–78)	70 (61–77)
Income (below 75k)	3,833 (53.2%)	1,314 (58.7%)	1,016 (64.8%)	518 (55.2%)	368 (55.6%)	2,200 (46.8%)	851 (60.1%)	20,157 (54.3%)
Insurance (yes)	7,142 (99.1%)	2,209 (98.7%)	1,537 (98.1%)	924 (98.4%)	652 (98.5%)	4,631 (98.5%)	1,394 (98.4%)	36,562 (98.5%)
Current daily cigarettes smoked (median, IQR)	0 (0–0)	0 (0–0)	0 (0–0)	0 (0–0)	0 (0–0)	0 (0–0)	0 (0–0)	0 (0–0)
Average daily cigarettes smoked (median, IQR)	0 (0–6)	0 (0–10)	0 (0–10)	0 (0–5)	0 (0–10)	0 (0–15)	0 (0–12.5)	0 (0–10)
Number of years smoked (median, IQR)	0 (0–10)	0 (0–20)	0 (0–21)	0 (0–10)	0 (0–18)	0 (0–17)	0 (0–20)	0 (0–15)
Smoking frequency (some or every day)	466 (6.5%)	242 (10.8%)	227 (14.5%)	62 (6.6%)	76 (11.5%)	358 (7.6%)	139 (9.8%)	3,403 (9.2%)
Electronic cigarette participant	490 (6.8%)	192 (8.6%)	157 (10.0%)	86 (9.2%)	55 (8.3%)	191 (4.1%)	114 (8.1%)	3,055 (8.2%)
Hookah participant	517 (7.2%)	174 (7.8%)	133 (8.5%)	97 (10.3%)	49 (7.4%)	279 (5.9%)	105 (7.4%)	3,310 (8.9%)
Cigar participant	1,702 (23.6%)	852 (38.1%)	590 (37.7%)	226 (24.1%)	260 (39.3%)	2,794 (59.4%)	588 (41.5%)	14,519 (39.1%)
Smokeless tobacco participant	172 (2.4%)	155 (6.9%)	133 (8.5%)	22 (2.3%)	53 (8.0%)	517 (11.0%)	110 (7.8%)	2,695 (7.3%)
Lung metastasis (present)	199 (2.8%)	149 (6.7%)	343 (21.9%)	48 (5.1%)	64 (9.7%)	88 (1.9%)	138 (9.7%)	1,012 (2.7%)
SNOMED code(s)	254,837,009	781,382,000	93,870,000	363,443,007	363,418,001	399,068,003	363,518,003	363,346,000 (excluding 363358000)

*The specific number of participants for counts less than 20 is not shown in accordance with *All of Us* Research Program policy. **All cancer except for primary lung cancer patients.

**Table 2:** Univariable and multivariable logistic regression analysis of continuous smoking-related variables in patients with lung metastasis versus patients without lung metastasis.

Condition	Univariable		Multivariable	
	Effect (95% CI)	p-value	Effect (95% CI)	p-value
Breast cancer (lung metastasis vs. no lung metastasis)				
Number of years smoked	− 0.01 (− 0.03, 0.00)	0.06	− 0.01 (− 0.03, 0.00)	0.09
Current cigarettes smoked per day	− 0.02 (− 0.06, 0.01)	0.27	− 0.03 (− 0.07, 0.01)	0.18
Average number of cigarettes smoked per day	− 0.02 (− 0.04, 0.00)	0.10	− 0.01 (− 0.03, 0.00)	0.18
Pack years	− 0.01 (− 0.03, 0.00)	0.17	− 0.01 (− 0.02, 0.00)	0.24
Liver cancer (lung metastasis vs. no lung metastasis)				
**Number of years smoked**	**− 0.01 (− 0.02, 0.00)**	**0.01**	− 0.01 (− 0.01, 0.00)	0.18
Current cigarettes smoked per day	− 0.01 (− 0.02, 0.01)	0.47	0.00 (− 0.02, 0.01)	0.78
Average number of cigarettes smoked per day	0.00 (− 0.01, 0.01)	0.59	0.00 (− 0.01, 0.01)	0.70
Pack years	0.00 (− 0.01, 0.00)	0.40	0.00 (− 0.01, 0.01)	0.94
Colorectal cancer (lung metastasis vs. no lung metastasis)				
Number of years smoked	0.00 (− 0.01, 0.01)	0.73	0.00 (− 0.01, 0.01)	0.89
Current cigarettes smoked per day	0.01 (− 0.02, 0.02)	0.63	0.00 (− 0.02, 0.02)	0.91
Average number of cigarettes smoked per day	0.00 (− 0.01, 0.02)	0.62	0.01 (− 0.01, 0.02)	0.41
Pack years	0.00 (− 0.01, 0.01)	0.86	0.00 (− 0.01, 0.01)	0.97
Ovarian cancer (lung metastasis vs. no lung metastasis)				
Number of years smoked	− 0.01 (− 0.03, 0.02)	0.67	− 0.01 (− 0.04, 0.02)	0.58
Current cigarettes smoked per day	− 0.02 (− 0.13, 0.04)	0.63	− 0.02 (− 0.13, 0.05)	0.64
Average number of cigarettes smoked per day	-0.01 (− 0.06, 0.02)	0.53	− 0.01 (− 0.06, 0.02)	0.46
Pack years	− 0.01 (− 0.05, 0.02)	0.52	− 0.01 (− 0.05, 0.02)	0.47
Pancreatic cancer (lung metastasis vs. no lung metastasis)				
Number of years smoked	0.00 (− 0.02, 0.01)	0.80	0.00 (− 0.02, 0.02)	0.92
Current cigarettes smoked per day	0.01 (− 0.02, 0.04)	0.48	0.01 (− 0.02, 0.04)	0.35
Average number of cigarettes smoked per day	0.01 (− 0.01, 0.03)	0.27	0.01 (− 0.01, 0.03)	0.14
Pack years	0.00 (− 0.01, 0.01)	0.56	0.01 (− 0.01, 0.02)	0.35
Prostate cancer (lung metastasis vs. no lung metastasis)				
Number of years smoked	0.00 (− 0.02, 0.01)	0.81	0.00 (− 0.02, 0.01)	0.77
Current cigarettes smoked per day	0.02 (− 0.01, 0.04)	0.17	0.02 (− 0.01, 0.04)	0.22
Average number of cigarettes smoked per day	0.01 (− 0.01, 0.02)	0.35	0.01 (− 0.01, 0.03)	0.26
Pack years	0.00 (− 0.01, 0.01)	0.59	0.00 (− 0.01, 0.01)	0.55
Renal cancer (lung metastasis vs. no lung metastasis)				
Number of years smoked	− 0.01 (− 0.02, 0.00)	0.11	− 0.01 (− 0.02, 0.01)	0.27
Current cigarettes smoked per day	− 0.02 (− 0.06, 0.01)	0.23	− 0.02 (− 0.05, 0.01)	0.32
Average number of cigarettes smoked per day	− 0.01 (− 0.02, 0.01)	0.49	0.00 (− 0.02, 0.01)	0.59
Pack years	− 0.01 (− 0.02, 0.00)	0.09	− 0.01 (− 0.02, 0.00)	0.14
All cancer (lung metastasis vs. no lung metastasis)				
**Number of years smoked**	**0.01 (0.01**,** 0.02)**	**< 0.01**	**0.01 (0.01**,** 0.02)**	**< 0.01**
**Current cigarettes smoked per day**	**0.01 (0.01**,** 0.02)**	**< 0.01**	**0.01 (0.00**,** 0.02)**	**< 0.01**
**Average number of cigarettes smoked per day**	**0.01 (0.00**,** 0.01)**	**< 0.01**	**0.01 (0.00**,** 0.01)**	**0.01**
**Pack years**	**0.01 (0.00**,** 0.01)**	**< 0.01**	**0.01 (0.00**,** 0.01)**	**< 0.01**

Significant values are in [bold].

Effect (beta) and p-value from logistic regression are shown. Multivariable models were adjusted for age, sex, income, and insurance status (sex not included for ovarian and prostate cancer analyses).

Analysis of binary smoking-related variables was performed next (Table 3). In the univariable analysis, an increased odds ratio of electronic smoke use in patients with lung metastasis was seen in the all cancer (OR = 1.38, 95% CI = 1.12–1.68, *P* < 0.01) and liver cancer (OR = 1.62, 95% CI = 1.12–2.32, *P* < 0.01) case groups with metastasis to the lung. Multivariable analyses demonstrated similar effects for both all cancer (OR = 1.29, 95% CI = 1.04–1.59, *P* = 0.02) and liver cancer (OR = 1.57, 95% CI = 1.06–2.28, *P* = 0.02) case groups. An increased odds ratio of cigar smoked participation (OR = 1.49, 95% CI = 1.06–2.08, *P* = 0.02) was observed in univariable analysis for colorectal cancer with metastasis. An increased odds of smoking frequency was also observed in both all cancer (OR = 1.34, 95% CI = 1.10–1.63, *P* < 0.01) and prostate cancer (OR = 2.02, 95% CI = 1.02–3.75, *P* = 0.03) case groups.

**Table 3:** Univariable and multivariable logistic regression analysis of smoking-related behaviors in patients with lung metastasis versus patients without lung metastasis in seven cancer types and a group of all patients with cancer (except primary lung cancer).

Condition	n (total)	Univariable		Multivariable	
		OR (95% CI)	p-value	aOR (95% CI)	p-value
Breast cancer (lung metastasis vs. no lung metastasis)					
Smoking frequency (some or every day)	7180	1.09 (0.60–1.83)	0.75	0.86 (0.47–1.45)	0.60
Electronic cigarette participant	7081	1.30 (0.76–2.10)	0.31	1.00 (0.57–1.63)	0.99
Hookah participant	7062	1.22 (0.71–1.96)	0.45	0.94 (0.54–1.56)	0.83
** Cigar smoke participant**	7059	0.75 (0.52–1.06)	0.12	**0.68 (0.46–0.97)**	**0.04**
Smokeless tobacco participant	7116	1.28 (0.50–2.69)	0.55	1.05 (0.40–2.24)	0.91
Liver cancer (lung metastasis vs. no lung metastasis)					
**Smoking frequency (some or every day)**	**1020**	**0.68 (0.47–0.98)**	**0.04**	0.74 (0.50–1.08)	0.13
**Electronic cigarette participant**	**1541**	**1.62 (1.12–2.32)**	**<0.01**	**1.57 (1.06–2.28)**	**0.02**
Hookah participant	1538	1.32 (0.87–1.96)	0.19	1.08 (0.70–1.66)	0.71
Cigar smoke participant	1541	0.93 (0.72–1.19)	0.58	0.98 (0.75–1.29)	0.90
Smokeless tobacco participant	1543	1.09 (0.70–1.64)	0.70	1.19 (0.75–1.83)	0.45
Colorectal cancer (lung metastasis vs. no lung metastasis)					
Smoking frequency (some or every day)	2232	0.92 (0.51–1.54)	0.75	0.76 (0.41–1.30)	0.34
Electronic cigarette participant	2212	1.68 (0.98–2.73)	0.05	1.37 (0.79–2.28)	0.24
Hookah participant	2195	1.60 (0.91–2.65)	0.08	1.21 (0.67–2.07)	0.50
**Cigar smoke participant**	**2191**	**1.49 (1.06–2.08)**	**0.02**	1.37 (0.95–1.97)	0.09
Smokeless tobacco participant	2207	1.43 (0.77–2.47)	0.22	1.14 (0.60–2.01)	0.68
Ovarian cancer (lung metastasis vs. no lung metastasis)					
Smoking frequency (some or every day)	933	1.30 (0.38–3.34)	0.63	1.36 (0.39–3.62)	0.58
Electronic cigarette participant	922	0.67 (0.16–1.88)	0.51	0.70 (0.16–2.05)	0.57
Hookah participant	916	0.59 (0.14–1.66)	0.39	0.63 (0.15–1.88)	0.46
Cigar smoke participant	921	0.65 (0.28–1.35)	0.28	0.67 (0.29–1.41)	0.33
Smokeless tobacco participant	924	0.00 (NA–1.3 × 10^14^)	0.99	0.00 (NA–6.8 × 10^13^)	0.99
Pancreatic cancer (lung metastasis vs. no lung metastasis)					
Smoking frequency (some or every day)	659	1.15 (0.49–2.40)	0.72	1.26 (0.52–2.77)	0.58
Electronic cigarette participant	648	1.20 (0.44–2.72)	0.69	1.13 (0.41–2.64)	0.80
Hookah participant	651	0.59 (0.14–1.67)	0.39	0.51 (0.12–1.51)	0.28
Cigar smoke participant	647	1.25 (0.74–2.12)	0.40	1.38 (0.77–2.44)	0.27
**Smokeless tobacco participant**	651	2.06 (0.90–4.27)	0.07	**2.33 (0.98–5.11)**	**0.04**
Prostate cancer (lung metastasis vs. no lung metastasis)					
**Smoking frequency (some or every day)**	**4683**	**2.14 (1.12–3.75)**	**0.01**	**2.02 (1.02–3.75)**	**0.03**
Electronic cigarette participant	4632	1.75 (0.67–3.73)	0.20	1.59 (0.61–3.47)	0.29
Hookah participant	4604	0.36 (0.06–1.16)	0.16	0.35 (0.06–1.11)	0.14
Cigar smoke participant	4617	0.73 (0.48–1.12)	0.14	0.74 (0.49–1.15)	0.18
Smokeless tobacco participant	4627	1.28 (0.66–2.28)	0.43	1.21 (0.62–2.16)	0.56
Renal cancer (lung metastasis vs. no lung metastasis)					
**Smoking frequency (some or every day)**	**1409**	**0.32 (0.11–0.72)**	**0.01**	**0.36 (0.12–0.82)**	**0.03**
Electronic cigarette participant	1388	1.32 (0.71–2.31)	0.35	1.31 (0.68–2.36)	0.40
Hookah participant	1388	1.24 (0.63–2.24)	0.50	1.10 (0.54–2.06)	0.78
Cigar smoke participant	1379	1.01 (0.71–1.45)	0.94	0.82 (0.55–1.20)	0.30
Smokeless tobacco participant	1387	1.51 (0.82–2.61)	0.16	1.26 (0.67–2.22)	0.45
All cancer (lung metastasis vs. no lung metastasis)					
**Smoking frequency (some or every day)**	**36,981**	**1.45 (1.20–1.75)**	**< 0.01**	**1.34 (1.10–1.63)**	**< 0.01**
**Electronic cigarette participant**	**36,524**	**1.38 (1.12–1.68)**	**< 0.01**	**1.29 (1.04–1.59)**	**0.02**
Hookah participant	36,358	1.02 (0.82–1.27)	0.79	0.95 (0.75–1.19)	0.67
**Cigar smoke participant**	36,356	0.93 (0.81–1.05)	0.25	**0.86 (0.75–0.99)**	**0.03**
Smokeless tobacco participant	36,576	1.13 (0.89–1.41)	0.29	1.02 (0.80–1.29)	0.85

Significant values are in [bold].

P-value from logistic regression shown. Multivariable models were adjusted for age, sex, income, and insurance status (sex not included for ovarian and prostate cancer analyses).

Multivariable analyses modeling electronic smoke use in patients with lung metastasis was repeated including an adjustment for the number of years smoked in the past or estimated cigarette pack years (Table 4). An increased odds of electronic smoke use in patients with liver cancer and lung metastasis was observed upon adjustment for the number of years smoked in the past (OR = 1.78, 95% CI = 1.14–2.75, *P* = 0.01) and the estimated cigarette pack years (OR = 1.60, 95% CI = 1.02–2.47, *P* = 0.04) but not for the all cancer group. The Spearman correlation between “some or every day” current smoking with e-cigarette use was higher in the all cancer group (ρ = 0.34) compared to liver cancer (ρ = 0.27) (Table 5).

**Table 4:** E-cigarette multivariable analysis adjusting for number of years smoked in the past, in addition to age, sex, income, and insurance status.

	Multivariable	
	aOR (95% CI)	p-value
Adjusted for number of years smoked in the past		
Breast cancer (lung metastasis vs. no lung metastasis)	1.05 (0.54–1.89)	0.88
**Liver cancer (lung metastasis vs. no lung metastasis)**	**1.78 (1.14–2.75)**	**0.01**
Colorectal cancer (lung metastasis vs. no lung metastasis)	1.54 (0.84–2.72)	0.15
Ovarian cancer (lung metastasis vs. no lung metastasis)	0.52 (0.08–1.93)	0.40
Pancreatic cancer (lung metastasis vs. no lung metastasis)	1.00 (0.31–2.74)	0.99
Prostate cancer (lung metastasis vs. no lung metastasis)	1.62 (0.53–4.05)	0.34
Renal cancer (lung metastasis vs. no lung metastasis)	1.76 (0.88–3.33)	0.09
All cancer (lung metastasis vs. no lung metastasis)	1.05 (0.83–1.33)	0.68
Adjusted for estimated cigarette pack years*		
Breast cancer (lung metastasis vs. no lung metastasis)	0.91 (0.45–1.69)	0.79
**Liver cancer (lung metastasis vs. no lung metastasis)**	**1.60 (1.02–2.47)**	**0.04**
Colorectal cancer (lung metastasis vs. no lung metastasis)	1.79 (0.98–3.13)	0.05
Ovarian cancer (lung metastasis vs. no lung metastasis)	0.61 (0.09–2.19)	0.51
Pancreatic cancer (lung metastasis vs. no lung metastasis)	1.03 (0.31–2.78)	0.96
Prostate cancer (lung metastasis vs. no lung metastasis)	1.48 (0.49–3.59)	0.43
Renal cancer (lung metastasis vs. no lung metastasis)	1.93 (0.94–3.71)	0.06
All cancer (lung metastasis vs. no lung metastasis)	1.15 (0.90–1.46)	0.25

Significant values are in [bold].

*Estimated cigarette pack years calculated by multiplying number of years smoked in the past by average cigarettes smoked per day divided by 20.

**Table 5:** Spearman correlation between “Some or every day” current smoking with e-cigarette use.

Cancer type	Spearman correlation	*p*-value
Breast cancer	0.31	< 0.01
Liver cancer	0.27	< 0.01
Colorectal cancer	0.31	< 0.01
Ovarian cancer	0.31	< 0.01
Pancreatic cancer	0.37	< 0.01
Prostate cancer	0.33	< 0.01
Renal cancer	0.26	< 0.01
All cancer	0.34	< 0.01

## Discussion

This study examines lung metastasis and smoking-related behavior in the AoURP and across a wide range of cancers. The all cancer case group results suggest that the number of years smoked, the current number of cigarettes smoked per day, the average number of cigarettes smoked, and estimated cigarette pack years have a weakly positive effect on lung metastasis. Additionally, in a multivariable model, electronic smoke use is associated with a higher odds ratio of lung metastasis in the all cancer and liver cancer case groups, which remains significant in liver cancer after adjustment for previous smoking history. This may be due to the higher correlation between current smoking and e-cigarette use in the all cancer case group compared to the liver cancer group. These results warrant further inquiry and suggest that both smoking and e-cigarettes may be associated with lung metastasis risk. These results may be explained by several possibilities. One possibility is that one or more chemical compounds in cigarettes and electronic cigarettes promote cancer metastasis and another is that participants who smoke cigarettes and develop cancer might switch to electronic cigarettes. The first possibility concurs with previously published findings in cellular models suggesting that e-cigarette use may promote lung metastasis^9^. The second possibility warrants further study. In the current dataset, we do not have temporal data about smoking or e-cigarette behavior over time, so future large cohort studies are needed to further evaluate this possibility.

Additional factors that may affect the electronic cigarette results include quantity, time, and type of e-cigarette used. Future prospective studies or detailed retrospective studies are needed to validate the results from this study and better characterize the effect of smoking and smoking-related behaviors on metastasis of non-lung cancers to the lung. Furthermore, wet lab studies are needed to better understand how cigarette and e-cigarette use may contribute to a lung PMN. To our knowledge, however, this is the first study characterizing the effect of smoking and smoking-related behaviors on lung metastasis in non-lung primary cancers.

The present study has several limitations, including variability in EHR data quality^13^, a limited sample size, lack of information on cancer stage, and the absence of a self-reported pack-year metric^14^ in the survey data for smoking behavior analysis, although an approximated pack-year metric was utilized as described in the methods. The variability in data quality was highlighted by the presence of a very small proportion of males assigned at birth with ovarian cancer and females assigned at birth with prostate cancer, suggesting imperfections in the data quality. Additionally, survey data may be subject to biases and subjectivity, and future work should validate the current study as new versions of the data are released and/or in an independent dataset. Taken together, the results from this study suggest that further research should be done to uncover the potential roles of cigarettes, nicotine, and electronic cigarette use in patients with cancer diagnoses, which may help guide future clinical management.

## Electronic supplementary material

Below is the link to the electronic supplementary material.

Supplementary Material 1

## Data Availability

The following data availability statement is included in the manuscript: “Data from this program are accessible at www.allofus.nih.gov, and this study was conducted on version 7 of the registered tier data utilizing the All of Us Researcher Workbench.”
