# Supplementary material for: Effects of smoking behavior on lung metastasis in the All of Us Research Program
Source: Sci Rep. 2025 Apr 1;15:11114. doi: 10.1038/s41598-025-89209-4 (PMC11962059; doi:10.1038/s41598-025-89209-4)
Supplement: Supplementary file 1 — Supplementary Material 1 [file 41598_2025_89209_MOESM1_ESM.docx]

Effects of smoking behavior on lung metastasis in the All of Us Research Program (Supplementary Materials)

**Survey Questions**

Lifestyle survey questions from the All of Us database can be found at this link: <https://www.researchallofus.org/wp-content/themes/research-hub-wordpress-theme/media/surveys/Survey_Lifestyle_Eng_Src.pdf>.

Additional survey questions from the All of Us database can be found at this link: <https://www.researchallofus.org/data-tools/survey-explorer/>

The survey questions are derived from previous survey-based studies, including the following:

- The Tobacco Use Supplement to the Current Population Study
- Million Veteran Program (MVP)
- Prostate, Lung, Colorectal, and Ovarian Cancer Screening Trial (PLCO)
- Population Assessment of Tobacco and Health (PATH) Study
- National Epidemiologic Survey on Alcohol and Related Conditions-III (NESARC-III)
- Instrument: AUDIT-C Questionnaire
- NIDA-Modified Alcohol, Smoking, and Substance Involvement Screening Test
